# Model-free phasor image analysis of quantitative myocardial T_1_ mapping

**DOI:** 10.1038/s41598-022-23872-9

**Published:** 2022-11-18

**Authors:** Wouter M. J. Franssen, Thomas A. Treibel, Andreas Seraphim, Sebastian Weingärtner, Camilla Terenzi

**Affiliations:** 1 Laboratory of Biophysics, Wageningen University and Research, Wageningen, The Netherlands; 2 Institute of Cardiovascular Science, University College London, London, UK; 3 Department of Cardiology, St Bartholomew’s Hospital, Barts Health NHS Trust, London, UK; 4 Department of Imaging Physics, Delft University of Technology, Delft, The Netherlands

**Keywords:** Biophysics, Cardiology, Medical research, Fluorescence imaging, Functional magnetic resonance imaging, Magnetic resonance imaging

## Abstract

Model-free phasor image analysis, well established in fluorescence lifetime imaging and only recently applied to qMRI ${T}_{2}$ data processing, is here adapted and validated for myocardial qMRI ${T}_{1}$ mapping. Contrarily to routine mono-exponential fitting procedures, phasor enables mapping the lifetime information from all image voxels to a single plot, without resorting to any regression fitting analysis, and describing multi-exponential qMRI decays without biases due to violated modelling assumptions. In this feasibility study, we test the performance of our recently developed full-harmonics phasor method for unravelling partial-volume effects, motion or pathological tissue alteration, respectively on a numerically-simulated dataset, a healthy subject scan, and two pilot patient datasets. Our results show that phasor analysis can be used, as alternative method to fitting analysis or other model-free approaches, to identify motion artifacts or partial-volume effects at the myocardium-blood interface as characteristic deviations, or delineations of scar and remote myocardial tissue in patient data.

## Introduction

Quantitative Magnetic Resonance Imaging (qMRI) has emerged as a promising technique for myocardial tissue characterization with a growing number of clinical applications^1^. Localized or global changes in relaxation time parameters (${T}_{1}$,${T}_{2}$,${T}_{2}^{*}$) have been shown to be useful clinical markers, specifically for focal or diffuse pathological tissue alterations in the myocardium^2–4^. Thus, relaxation time mapping, i.e. the voxel-wise quantification of relaxation times, is increasingly more established in clinical use^2^.

Native myocardial ${T}_{1}$ mapping is most commonly used in cardiovascular MRI, and is a recommended part of the clinical work-up for a number of disease entities, including amyloidosis, Anderson-Fabry disease and myocarditis^2,5–7^. Modified Look-Locker Inversion Recovery (MOLLI)^8^ is most commonly used for cardiac ${T}_{1}$ measurements, and enables sampling the Inversion Recovery curve in a single breath-hold. For MOLLI data, mono-exponential fitting is conventionally used to obtain ${T}_{1}$ maps, although it is well known that qMRI data with either multi- or non-exponential character may be incorrectly described^9,10^. Yet, bi-exponential fitting typically leads to large processing errors when applied to data with limited signal-to-noise ratio (SNR) such as clinical ${T}_{1}$ maps.

Furthermore, data corruption induced by, e.g., motion may lead to a large deviation from the fitting model, resulting in non-robust results^11,12^. Multiple techniques for motion correction have previously been proposed^11–14^. However, it has been suggested that identifying motion during the scan and reacquiring the maps, if needed, is a more effective way to overcome motion challenges^15^. To this end robust and versatile methods of motion detection are highly desirable.

In this work, we explore the performance of a robust, fitting-free, lifetime image processing method, called phasor analysis, for unravelling partial-volume effects, motion or pathological tissue alteration in myocardial ${T}_{1}$ qMRI data. The phasor method was originally developed for, and is nowadays routinely used in, fluorescence lifetime imaging (FLIM) studies, as well as in diffusion mapping of fluorescent species and multi-dimensional analysis of hyperspectral super-resolution imaging data^16–19^. Very recently this method has been adapted^20^, and further optimized by some of us^21^, for the analysis of ${T}_{2}$ and diffusion qMRI relaxometry data from a range of systems, including bio-medical tissues^22^. Phasor relies on the mathematically-exact Fourier Transform (FT) analysis of all the measured signal decays within the image and, thus, provides information about the distribution of lifetimes across the voxels without resorting to fitting procedures. In conventional phasor analysis, only the first or second Fourier coefficient are typically used, e.g. in FLIM studies, as they contain the highest signal intensity. The real and imaginary parts of such Fourier coefficients are then displayed in a two-dimensional graph, called ‘phasor plot’, for all image voxels simultaneously^17^. Such phasor plot enables straightforward identification of signal decays for all image voxels. Mono-exponential behaviours are readily identified by the clustering of datapoints along a reference semicircle curve. Datapoints in the phasor plot, which lie below this reference mono-exponential curve, refer to voxels with multi-exponentially decaying signals. The distribution of such datapoints is key to unravelling the number of exponential terms present in the signal decays across the image. In our recently developed full-harmonics approach, where all Fourier coefficients are conjointly retained for constructing the phasor plot, the unmixing accuracy of phasor analysis for qMRI data with multi-modal decays was shown to be maximized as compared to single first- or higher-harmonics approaches typically adopted in FLIM studies^21,23^. This improvement turned out crucial for boosting the ability of phasor representation of qMRI data to unravel *inter-*voxel trends across the image such as, e.g., partial-volume effects. In phasor analysis, all the above-mentioned information is achieved without introducing any fitting error or model assumption, and can be exploited as prior knowledge for any subsequent image analysis step, iterative or non. For instance, we have shown that phasor-coordinate images can be reconstructed and yield consistent information as compared to qMRI relaxation maps obtained by fitting analysis^21^.

In this paper, we evaluate the use of full-harmonics phasor processing for the analysis of cardiac ${T}_{1}$ mapping data, and for the first time we apply this method to the identification of motion corruption and of pathological tissue alterations. To these scopes, in analogy to the approach adopted for characterizing partial-volume effects^21^, we exploit either or both phasor-coordinate images, to be directly compared with ${T}_{1}$ maps, and phasor plot representations, where deviations from neat uni- or multi-modal signal decay behaviours are readily identified. First, numerically simulated data are used to explore the effect of partial-voluming and motion-corruption in phasor analysis. Secondly, cardiac ${T}_{1}$ mapping data from healthy subjects are used to validate such phasor characterization *in-vivo*. Finally, pilot patient phasor data are obtained to evaluate the ability of our proposed image analysis method to discern diseased myocardial tissue.

## Materials and methods

### Phasor processing

Phasor processing of qMRI relaxation data can be used to map the signal decay from each image voxel to a lifetime value on a 2D phasor plane. Such conversion is obtained, per voxel, by the FT of the respective measured decay along the sampled contrast dimension, e.g. given by the number of echoes in ${T}_{2}$ mapping or by the inversion time (TI) in ${T}_{1}$ mapping. As explained in detail elsewhere^17,21^, all datapoints in each FT curve of the image are normalized to the respective real value of the zeroth-order Fourier coefficient of the FT dataset. The latter value in turn corresponds to the measured equilibrium ^1^H NMR signal intensity, or integral area of the measured signal decay over the contrast dimension. The set of sampled Fourier coefficients, with their real and imaginary parts, can be used to define two suitable variables that are plotted against each other in the phasor plot. Traditionally, only the real and imaginary parts of the first Fourier harmonic, here respectively denoted as Re_1_ and Im_1_, are used in FLIM phasor studies^17^. Some of us have recently shown that a more accurate mapping of the phasor-space information to a 2D plot is achieved when using all Fourier coefficients, or ‘harmonics’, simultaneously^21^. This approach, called full-harmonics phasor analysis, requires handling a $N-$ dimensional phasor-space data, where $N$ is the number of measured Fourier harmonics, and hence defining a two-dimensional projection phasor plane based on the selection of three lifetimes. In the case of bi-exponential data examined in the present work, these values are chosen to correspond to the observed ${T}_{1}$ values, and to include a third in-between ${T}_{1}$ value, with the aim of maximizing the distance between the bi-exponential line of clustered data points in the phasor plot and the reference semi-circle^21^. We note that input-free principal component analysis (PCA), which also selects as projection axis the one where the largest variance occurs, could be used as well for constructing full-harmonics phasor plots. Yet, as previously demonstrated by some of us for the case of bi-exponential data^21^, of interest for the present application to cardiac ${T}_{1}$ mapping, while the separation of the two mono-exponential positions is still optimized when using PCA, the distance to the semicircle may be sub-optimal. This is because the semicircle is not considered during PCA analysis. The full-harmonics phasor algorithm, also used in the present work, has been previously described in detail and already successfully validated^21^. We here report, in “Choice of projection plane in full-harmonics phasor analysis” of Supplementary Information, the calculation steps needed for choosing a projection plane: in this work this was, for the above-mentioned reason, based on the choice of three lifetimes values rather than on PCA calculations.

The specific position of a point in the phasor plot depends on the lifetime and multi-modal character of the corresponding signal decay function. In ^1^H qMRI data, the uncertainty in the calculation of the phasor coordinates depends on the signal-to-noise ratio of the MRI image, where the per-voxel signal intensity depends, among other factors, on the respective number of ^1^H atoms, on the voxel size and on the sensitivity of the MRI scanner^24^. Because the phasor coordinates of all voxels in the imaging dataset are plotted simultaneously, voxels with similar relaxation decay properties will lead to clusters of data points. Distinguishing such clusters is key to enabling the identification of different tissues, with distinct lifetimes, in a model- and fitting-free way.

For visualizing these clusters, a reference curve, also called ‘semicircle’, is generally displayed within the phasor plot. This reference curve is obtained by plotting the phasor coordinates of image voxels whose signal decays are single-exponential decays, with lifetimes chosen over a range wide enough to represent the measured data. In such phasor representation, image voxels whose phasor coordinates lie along the reference curve are unequivocally described by a mono-exponential decay. In general, image voxels with multi-exponential decays fall below the phasor reference semicircle, while those with non-exponential, e.g. gaussian, decay functions fall above such reference curve. A useful property of the phasor plot representation is that image voxels with bi-exponential signal decays yield points that fall along a straight line connecting the phasor coordinates, along the semicircle, of the two respective lifetimes. This is the case of image voxels with partial-volume effects, i.e. containing overlapping signals from two distinct tissue types. Datapoints from such voxels appear continuously distributed along a line below the semicircle, as a consequence of the varying signal intensity ratios between the two tissues. As described elsewhere^20,21^, three- or four-exponential decays respectively lead to triangular- or rectangular-shaped clouds of phasor-space coordinate values.

Conventional phasor processing, as adopted in FLIM, requires the imaging signal to exponentially decay as a function of a contrast parameter. In qMRI measurements, this occurs in the case of both ${T}_{2}$ relaxation and diffusion data. Indeed, these types of qMRI data have already been successfully investigated by phasor^20–22^. In the present work, we set out to validate the use of phasor also for Inversion Recovery ${T}_{1}$ data, where signal intensities instead follow an exponentially increasing function. For the purpose of phasor analysis, such type of data must be re-shaped in order to obtain time-decaying behaviours. To convert the exponential recovery to an exponential decay, amenable to phasor processing, the sign of the whole measured signal intensity dataset must be inverted, and the asymptotic signal needs to be subtracted. In this work, where a MOLLI ${T}_{1}$ mapping method was used, the data point sampled with the longest inversion time was used as an approximation of the asymptote. In MOLLI, the longest inversion time is typically multiple times longer than the expected ${T}_{1}$, leading to a fair approximation of the asymptote. In other commonly used ${T}_{1}$ mapping techniques based on saturation recovery^25,26^, an image without magnetization preparation is commonly acquired. This can be used to obtain an exponential decay, when processing saturation-based myocardial ${T}_{1}$ mapping techniques. The adjusted qMRI ${T}_{1}$ dataset can then be processed by phasor. We note that, if the mono-exponential phasor reference curve is reconstructed in this way, all the above-mentioned useful characteristics of a phasor plot are retained^10,11^.

### Numerical simulations

A numerical phantom of the myocardium was created to resemble the left ventricle in short-axis view, by simulating two exponential signal decays for two distinct areas, namely: an outer ring, mimicking the myocardial tissue, and its inner circular area, corresponding to the signal from blood. The signal intensity for voxels outside the simulated phantom was fixed to zero. The equilibrium value of the ^1^H MRI signal intensity per unit volume was normalized to 1 for both myocardium and blood regions. Using this definition, a 1000 × 1000 grid of voxels was generated and subsequently down-sized to a 21 × 21 grid to simulate partial-volume effects. For each voxel within the down-sized data, with known myocardium and blood signal intensities, a bi-exponential signal decay was simulated using ${T}_{1}$ values of 1500 and 2000 ms for myocardium and blood, respectively. The sampled $TI$ time points were 129, 209, 1344, 1394, 2494, 2551, 3644, and 4807 ms, for comparison with the measured dataset discussed in “Partial-volume effects” section. An offset correction was performed, as described in “Phasor processing” section. The full-harmonics phasor projection plane was defined by using three ${T}_{1}$ values, namely 1.5, 1.75, and 2 s^21^. Details about the calculation of the full-harmonics phasor projection plane are provided in Section “Choice of projection plane in full-harmonics phasor analysis” of Supplementary Information, as well as in our previous paper^21^.

The position of data points in the phasor plot can be converted to a myocardium volume fraction by comparing the observed position with a pre-calculated library of positions for all possible volume fractions, using these specific ${T}_{1}$ values for blood and myocardium muscle respectively. The ${S}_{myo}/{S}_{tot}$ value that, in the phasor plot, lies closest to the experimental point is then selected as the ${S}_{myo}/{S}_{tot}$ value for this experimental point.

Segmentation of the simulated imaging data into three distinct areas, namely myocardium, blood, and regions with partial-volume effects, was obtained by using the $x-$ coordinate of the phasor plot, namely $Axis1$, respectively using $0.88>Axis1>0.85$ for myocardium, $0.85>Axis1>0.43$ for partial-volume voxels, and $0.43>Axis1>0.41$ for blood. These $Axis1$ ranges were manually chosen by visual inspection of the phasor coordinates at the ends of the bi-exponential cluster of datapoints, on the reference phasor curve. We note that automating the selection of $Axis1$ ranges, while desirable for future potential clinical applications of phasor-based segmentation, was not explored in the present feasibility phasor study of qMRI ${T}_{1}$ data.

### Healthy subjects

Patients and controls were recruited under ethics approved by the ethical committee of UK National Research Ethics Service (07/H0715/101), conforming to the principles of the Helsinki Declaration, and all subjects gave written informed consent.

#### Partial-volume effects

Healthy subject data was acquired for one volunteer (male, 28 years old) using the MOLLI pulse sequence^8^ on a 3 T MRI scanner (Magnetom Skyra; Siemens Healthineers, Erlangen, Germany) with the following imaging parameters: balanced Steady-State Free-Precession image acquisition (bSSFP); TR/TE/α = 2.6 ms/1.0 ms/35°, in-plane resolution = 1.7 × 1.7 mm^2^, slice-thickness = 6 mm, field-of-view = 440 × 375 mm^2^, bandwidth = 1085 Hz/px, number of $k$-space lines = 139, linear profile ordering, startup-pulses = 5 Kaiser-Bessel, GRAPPA-factor = 2. The 5(3 s)3 MOLLI scheme was employed for native ${T}_{1}$-mapping. The sampled $TI$ time points were 129, 209, 1344, 1394, 2494, 2551, 3644, and 4807 ms.

Regions-of-interest (ROIs) for phasor analysis were assigned based on the last intensity image of the series, and the myocardium ring and the voxels within this were selected. Segmentation of the measured imaging data was performed according to the $Axis1$ intervals used for the simulated data (see “Numerical simulations” section).

#### Measured and simulated motion-corruption effects

A second set of healthy subject data was acquired on a 26 years old man using MOLLI on a 3 T MRI scanner (Ingenia; Philips, Best, Netherlands). The following imaging parameters were used: balanced Steady-State Free-Precession image acquisition (bSSFP); TR/TE/α = 2.3 ms/1.1 ms/20°, in-plane resolution = 2.0 × 2.0 mm^2^, slice-thickness = 8 mm, field-of-view = 280 × 280 mm^2^, bandwidth = 1082 Hz/px, number of $k$-space lines = 90, linear profile ordering, startup-pulses = 10 linear sweep-up, SENSE-factor = 2. The 5 s(3 s)3 s MOLLI scheme was employed for native ${T}_{1}$-mapping. The sampled $TI$ time points were: 136, 350 , 1080 , 2034, 2171, 2969, 3094, 4762 ms for data acquired during breath-holding, or 136, 350 , 1059, 1213, 2021, 2123, 2924, 3056, 3811, 4694 ms for data acquired during during free-breathing. Such data were collected in order to study the effect of respiratory-induced motion artifacts.

In order to mimic a sudden movement between subsequent signal acquisitions with the MOLLI sequence, simulated motion was artificially added to the breath-hold measured dataset by shifting along the vertical spatial direction, by 8 voxels (9.3 mm), the voxels in the MRI images corresponding to the even-numbered inversion time values. As a region-of-interest (ROI), the ring of myocardium tissue and its inner region were selected based on the image corresponding to the fourth inversion time, namely 1213 ms. This image was selected for its highest visual contrast between myocardium and blood, and the definition of the ROI is shown in Figure S1 of SI. The selected ROI was symmetrically broadened by 10 pixels along all directions, to accommodate also all motion-shifted voxels.

### Patients

Patient data were acquired in two subjects with obstructive coronary artery disease and evidence of myocardial infarction (57 and 76 years old, both male). Patient imaging was performed at 1.5 T (Magnetom Aera; Siemens Healthineers, Erlangen, Germany). Identical image parameters to the subjects in “Partial-volume effects” section were used except for the following: TR/TE/α = 2.6 ms/1.1 ms/35°, in-plane resolution = 1.7 × 1.7 mm^2^, slice-thickness = 8 mm, field-of-view = 430 × 322 mm^2^, bandwidth = 1085 Hz/px, number of $k-$ space lines = 125, linear profile ordering, startup-pulses = Kaiser-Bessel, GRAPPA-factor = 2, 5(3 s)3 MOLLI scheme. The sampled $TI$ time points were: 100, 180, 860, 942, 1617, 1700, 2372, 3127 ms for data shown in the top row of Fig. 4, or 100, 180, 1202, 1225, 2282, 2282, 3370, 4440 ms for data shown in the bottom row of Fig. 4. Additionally, conventional 2D PSIR Late Gadolinium Enhanced (LGE) Images were acquired for reference.

The ROIs for phasor analysis were assigned based on the respective images acquired at the longest inversion time, and are shown in Figure S2 of SI alongside the respective labelling of healthy and scarred tissues. Segmentation of the images shown in “Patients: identification of scarred myocardial tissue” section was done according to the following intervals, respectively for the first or second volunteer: $0.65>Axis1>0.56$ or $0.74>Axis1>0.62$ for myocardium, $0.56>Axis1>0.44$ or $0.62>Axis1>0.54$ for scar tissue voxels, and $0.44>Axis1>0.35$ or $0.54>Axis1>0.40$ for blood.

## Results

### Numerical simulation of cardiac T_1_ data

**Figure 1:**
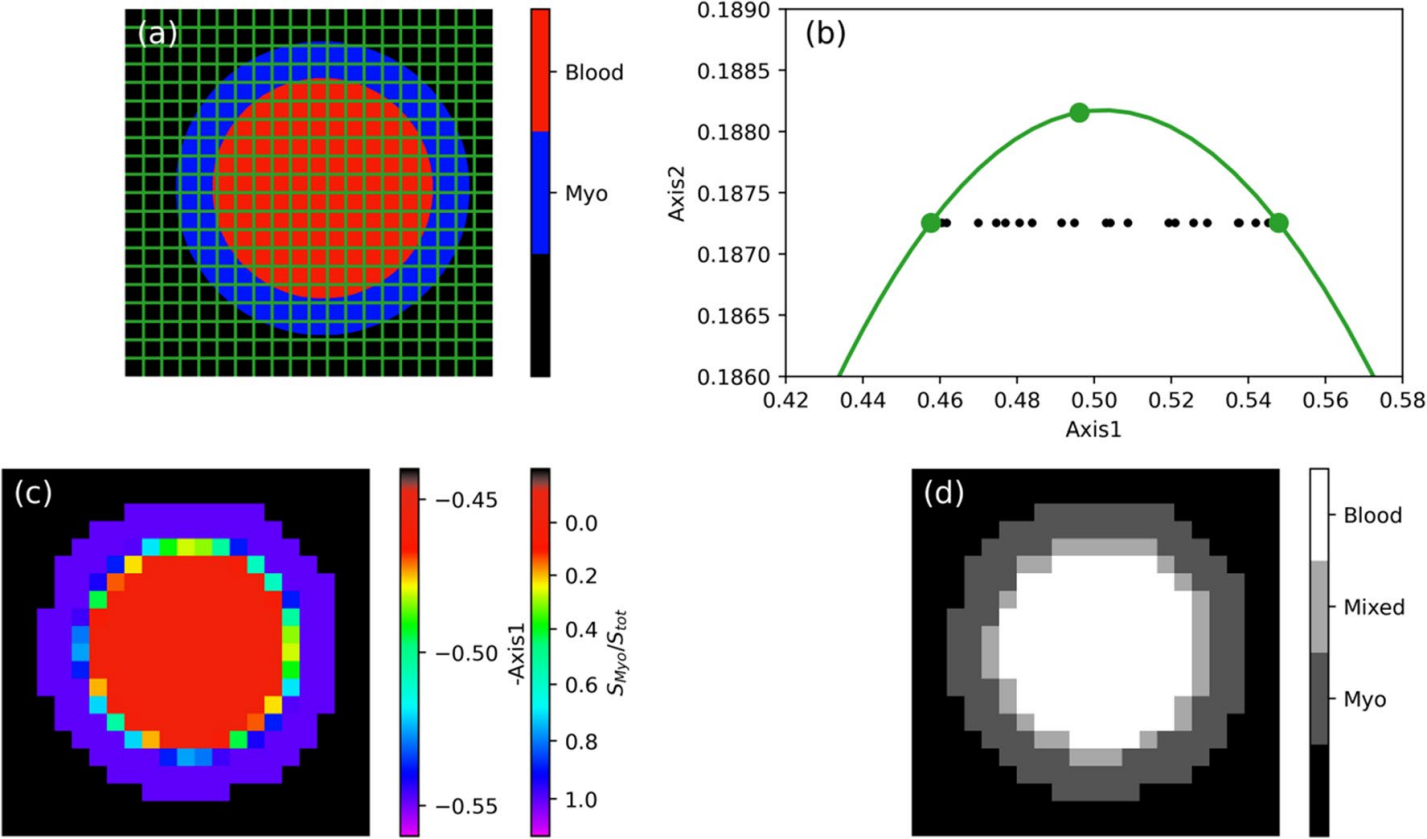
Phasor processing of ${T}_{1}$ qMRI data from a simulated heart phantom. (**a**) Sample architecture, indicating the used sampling grid, with the blood pool in red, and the myocardium in blue. The outside region in black corresponds to absence of MRI signal. (**b**) Full-harmonics phasor plot of the Inversion Recovery data simulated for the phantom in (**a**). In green, the mono-exponential reference curve and the dots referring to the three positions used for the full-harmonics projection with ${T}_{1}$= $1.5$, $1.75$ and $2$ s^21^. (**c**) and (**d**) Image reconstructed from the phasor-space coordinates and its corresponding phasor-based segmentation, respectively.

We first demonstrate the validity of our full-harmonics phasor processing method on a virtual heart phantom, schematically depicted in Fig. 1a, for which ${T}_{1}$ mapping data were numerically simulated. This phantom was designed to contain voxels with individual ${T}_{1}$ values from either blood (red) or myocardium (blue), as well as voxels containing both compartments.

Figure 1b shows the resulting phasor plot for this simulated dataset obtained by using the full-harmonics method^21^. We underline that such phasor representation was obtained without any assumption on the number and value of the lifetimes present. The data points (filled black circles) from all voxels in Fig. 1a fall along a straight line connecting two points (filled green circles) along the reference phasor curve (solid green line). Such phasor-space points, correspond to the ${T}_{1}$ values for myocardium (rightmost point) and blood (leftmost point) ^1^H signals, respectively. Data points distributed continuously along the straight line connecting these two points represent all voxels in the simulated qMRI dataset that contain *both* blood and myocardial ^1^H ${T}_{1}$ values, in varying relative fractions of tissue and blood.

The existence of voxels with signal from both myocardium and blood can be directly visualized by reconstructing an image of the virtual heart phantom using, as contrast intensity scale, the corresponding phasor-space coordinate, $Axis1$, in Fig. 1b. Such phasor-space coordinate corresponds to the myocardium volume percentage ${S}_{myo}/{S}_{tot}$ (see “Numerical simulations" section). In this phasor-based image (Fig. 1c), voxels which contain both myocardial and blood compartments are coloured in-between. From such an image, a segmentation map can be obtained (Fig. 1d) where the net blood (white) or tissue (dark grey) environments are clearly distinguished from voxels containing both compartments (light gray).

### Healthy subjects: partial-volume or motion-corruption effects

**Figure 2:**
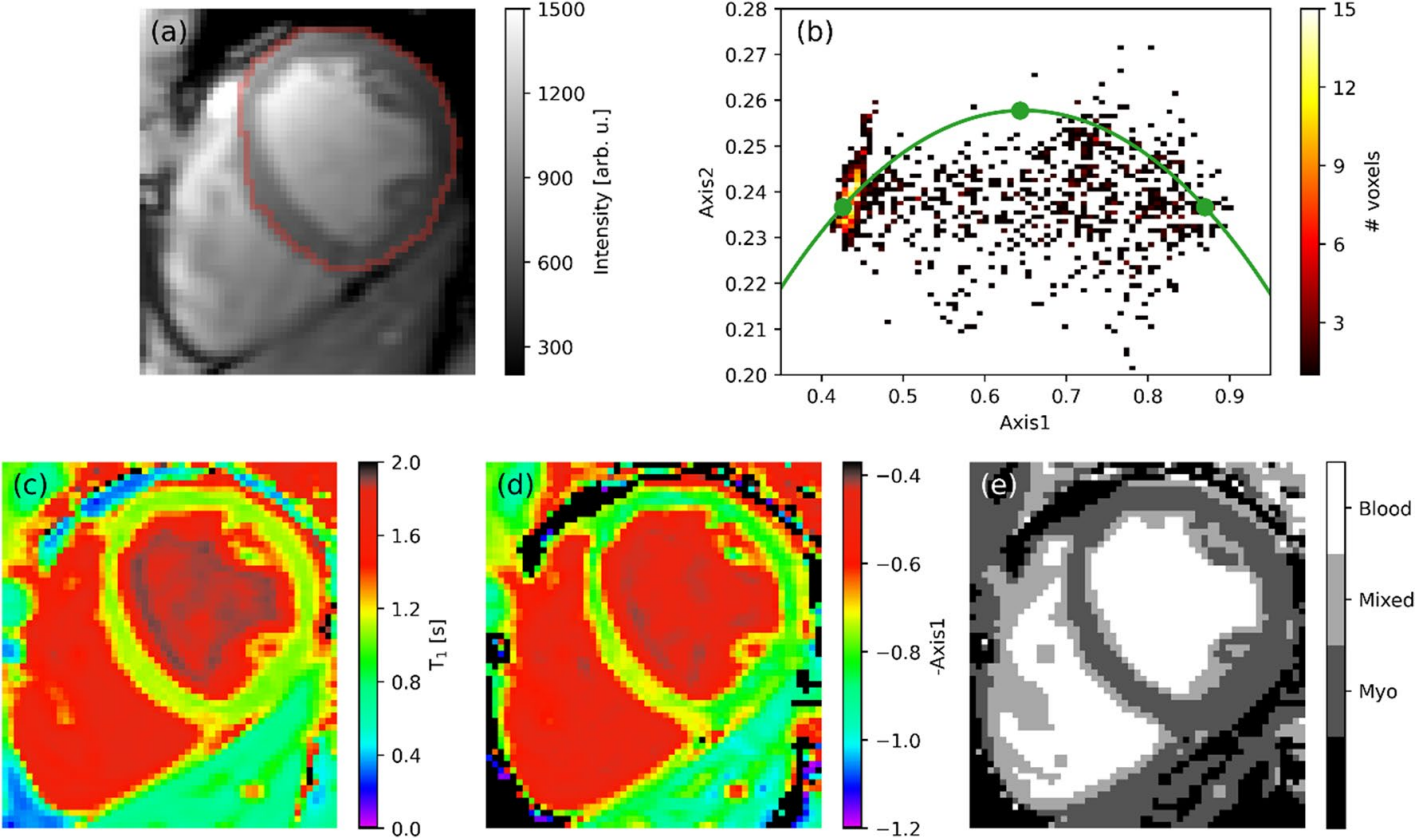
Phasor or fitting analysis of a ^1^H ${T}_{1}$ qMRI dataset recorded on a healthy volunteer using a MOLLI pulse sequence. (**a**) Intensity map obtained from the first TI value of the MOLLI data. In red, the outline of the region selected for the phasor analysis is shown. (**b**) Full-harmonics phasor plot of the selected region. In green the mono-exponential reference curve is plotted, with the green dots referring to the three positions used for the full-harmonics projection^21^. (**c**) ${T}_{1}$ map obtained by single-exponential two-parameter fit. (**d**) Phasor-coordinate map derived from the $x$-axis coordinate of the phasor plot, where the phasor-coordinate colourmap has been inverted to match the colour scheme of the ${T}_{1}$ map in (**c**). (**e**) Map of the phasor-based segmentation.

Figure 2 demonstrates the result of full-harmonics phasor analysis on MOLLI ${T}_{1}$ data acquired on a healthy individual. Figure 2a shows the MRI image acquired at the longest inversion time, extracted from the ${T}_{1}$ data, where the ROI selected for further phasor analysis is highlighted. In the phasor plot of such data (Fig. 2b), the blood (left) and myocardium (right) signal clusters are connected by a straight band of data points that lies below the mono-exponential reference phasor curve, in analogy with what observed for the simulated heart phantom (Fig. 1b). The larger data dispersion around the straight line, as compared to the numerical simulation results shown in Fig. 1b, is due both to the presence of noise, not included in the simulations of Sect. Numerical simulation of cardiac T_1_ data, and to the larger structural heterogeneity in the measured cardiac data.

Figure 2c shows the ${T}_{1}$ map obtained by single-exponential two-parameter fitting of the data. Figure 2d,e respectively show the phasor-coordinate map and its corresponding segmentation results. In analogy to Fig. 1d for the simulated phantom, Fig. 2e enables spatially identifying voxels (light grey) with co-existence of blood (white) and myocardial (dark grey) ${T}_{1}$ components.

**Figure 3:**
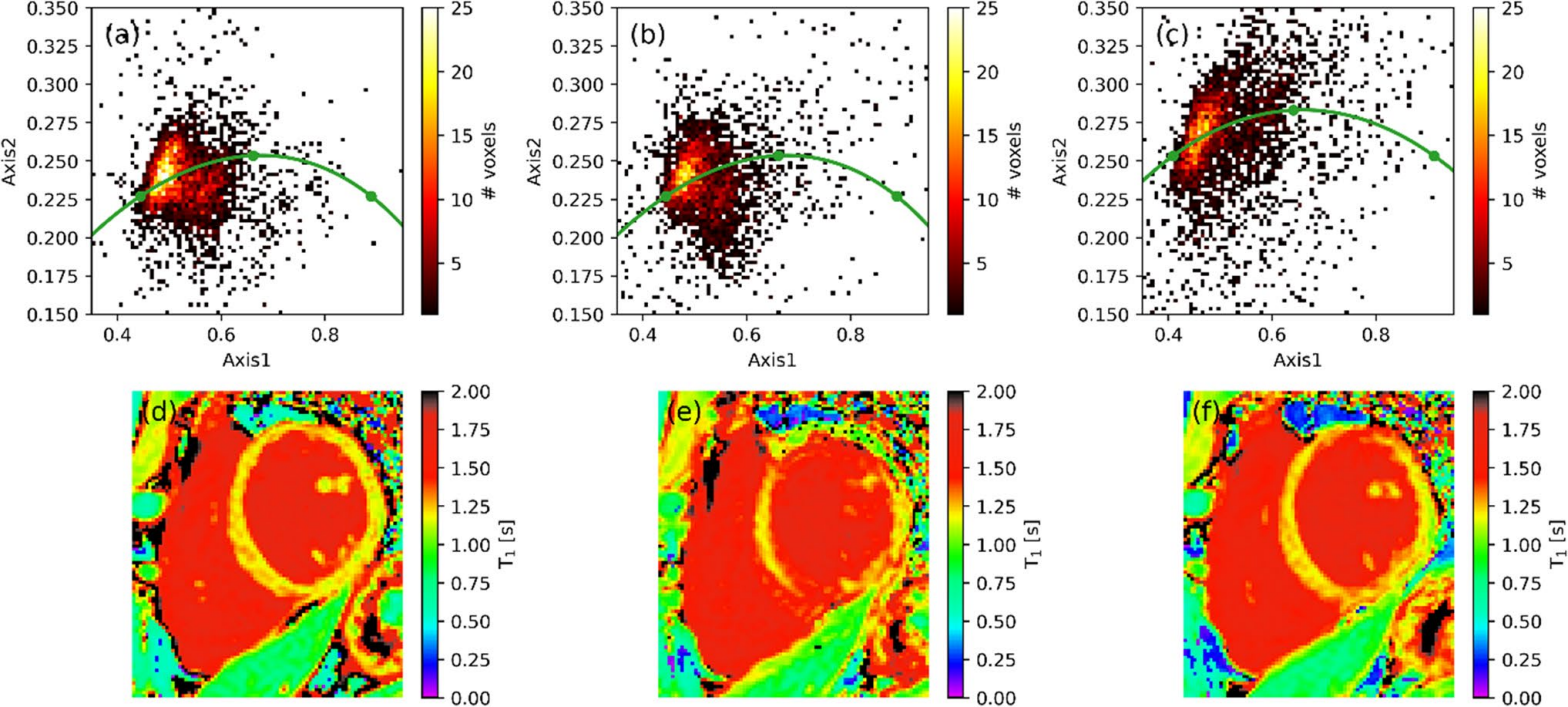
Effect of motion on full-harmonics phasor plots, with their reference curve in green (top), and on single-exponential ${T}_{1}$ mapping images (bottom) for ^1^H qMRI ${T}_{1}$ data for a healthy volunteer. The columns refer to data either devoid of motion artifacts (left) or with added simulated motion using voxel shift (middle), and to data recorded during free breathing of the volunteer (right). The projection plane of the phasor plots was defined by three ${T}_{1}$ values, namely 0.7, 1.1, 2 s. The reference curve in (**c**) looks different than in (**a**) and (**b**) due to both the different data sampling (see Sect. “Measured and simulated motion-corruption effects”) and the additional imaging noise caused by respiratory motion.

In Fig. 3a demonstration is shown of the performance of phasor analysis for the investigation of motion-induced corruption artifacts in ${T}_{1}$ mapping images. To this scope, phasor plots and ${T}_{1}$ maps for data acquired on a healthy volunteer in the absence of motion artifacts (Fig. 3a,d) are compared with the results obtained either from the same dataset, by artefactually shifting the image voxels as described in Sect. “Measured and simulated motion-corruption effects” (Fig. 3b,e), or from data acquired, on the same healthy volunteer, under free-breathing conditions (Fig. 3c,f). As shown for the latter case, the presence of motion artifacts can yield an increased scatter of data points, in the phasor plots, along the direction perpendicular to the bi-exponential cloud which in turn, for the present dataset, lies very close to the reference phasor curve.

### Patients: identification of scarred myocardial tissue

**Figure 4:**
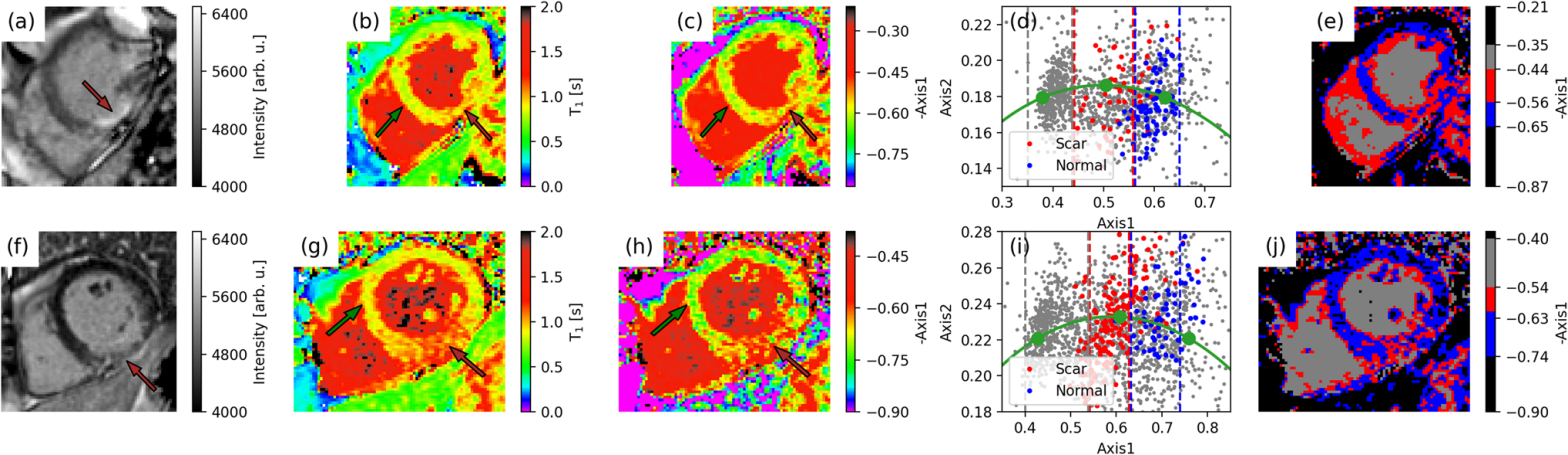
For two diseased individuals (**a**–**e** or **f**–**j**): phasor or fitting analysis of MOLLI data. (**a**, **f**) LGE images, with the scar indicated by the brown arrow; (**b**,**g**) ${T}_{1}$ and (**c**,**h**) phasor-coordinates images; (**d**,**i**) phasor plots voxels identified either as scar muscle depicted in red, or as healthy myocardium tissue plotted in blue with the reference phasor curve in green The brown and green arrows in the phasor and ${T}_{1}$ maps indicate the regions, fully shown in Figure S2 of SI, where these phasor plot voxels are taken from. The data points in grey refer to the whole phasor-coordinate images, partly overlapping with data points for healthy and scar tissue. (**e**, **j**) Phasor-based segmentation images for the three phasor plot regions, defined based on $Axis1$ intervals marked in the phasor plots (**d**, **i**) as dashed lines referring to healthy (blue) and scar (red) myocardial tissue, as well as to the whole image (gray) (see Sect. “Patients”). The black regions in (**e**, **j**) refer to image voxels, also plotted in gray in (**d**, **i**), falling outside the three selected $Axis1$ intervals and attributed to background noise.

To further illustrate the applicability of phasor processing to the analysis of ${T}_{1}$ mapping data of myocardium, we have examined the images from two different diseased patients. Both patients exhibit sub-endocardial scarring. Figure 4 shows the comparison of the hyperintense scar areas in LGE images (Fig. 4a,f) with the corresponding ${T}_{1}$ (Fig. 4b,g) or phasor-coordinates (Fig. 4c,h) images. In the latter two images, two ROIs are marked with an arrow: namely, an area of normal myocardium (green) and that for the scarred tissue (brown). In the respective phasor plots (Fig. 4d,i), on top of the whole set of data points corresponding to the phasor-coordinate images (grey filled circles), also the datapoints corresponding solely to either the normal (blue filled circles) or the scarred (red filled circles) tissue are highlighted. In blue and red, the regions referring to either the normal myocardium or to the scarred tissue, as identified in the phasor plots (Fig. 4d,i), are shown also on the respective phasor-based segmentation images (Fig. 4e,l), alongside the voxels in gray referring to the whole phasor-coordinate maps. The intervals chosen for the latter, $Axis1$-based, image segmentation are displayed in the phasor plots (Fig. 4d,i) and described in Sect. “Patients”.

## Discussion

This work aimed at showing the performance of phasor analysis as alternative, non-fitting, qMRI image processing method for applications to ${T}_{1}$ mapping data from healthy or diseased myocardial tissues. To this end, we have targeted (i) the identification of partial-volume effects and motion-induced artifacts in cardiac ${T}_{1}$ data in the absence of a disease, and (ii) the delineation of pathological tissue in pilot patient data. We have tested a recently introduced full-harmonics phasor analysis, which has been recently validated for the characterization of multi-exponential ${T}_{2}$ qMRI decays^20,21^. This method has been shown to maximize the unmixing accuracy as compared to conventional, single-harmonics, phasor analysis. In the present work, our optimized full-harmonics phasor processing approach has been further adjusted for the analysis of ${T}_{1}$ qMRI datasets, by introducing baseline correction and sign adjustment in order to transform the acquired dataset into a signal decay similar to that of ${T}_{2}$ relaxation. As discussed in “Phasor processing” section, this data conversion step is needed in order to apply the FT analysis to ${T}_{1}$ qMRI datasets and obtain the corresponding phasor plots.

Our first demonstration in Fig. 1 concerned numerically simulated ${T}_{1}$ data for a virtual myocardium phantom, containing voxels with blood or myocardial tissue only, as well as voxels with both components. Full-harmonics phasor plots indicate that there are two clusters of voxels with mono-exponential ${T}_{1}$ character, which fall along the phasor reference curve. These clusters of voxels correspond to the signal from either myocardium tissue or blood. In addition, there is a set of voxels at the interface between these two regions that exhibit partial-volume effects. These voxels yield phasor data points lying along a straight line that intersects the mono-exponential reference curve in correspondence of the lifetimes of myocardium or blood signal decays. The position of each voxel along such line depends on its specific myocardium-to-blood signal intensity ratio. We note that, in the absence of partial-volume effects, only two isolated clusters of data points would be observed, respectively centred around the phasor coordinates for either myocardium or blood individual lifetimes.

From the phasor plot in Fig. 1b, we have obtained an image of the phasor-space coordinates, indicative of the myocardium volume fraction. The latter image was further processed for reconstructing a segmented image based on phasor analysis (Fig. 2e). The same image analysis approach has been applied to analyse a ${T}_{1}$ mapping dataset collected from a healthy volunteer in the absence of motion artifacts (Fig. 2). Results are consistent with the simulations shown in Fig. 1. Also for this in vivo dataset, the phasor plot in Fig. 2b shows clear evidence of partial-volume effects between myocardium and blood signal pools, with the latter two populations being separated by a factor of about 2 along the $Axis1$ phasor coordinate. We note that, as compared to the simulated data in Fig. 1b, for the real case study a wider scatter of data points around the straight line exists due to that in Fig. 2b both noise and structural heterogeneities are present. From this first validation of phasor analysis on both simulated and measured ${T}_{1}$ mapping data for a healthy myocardium, we conclude that our adjusted phasor processing method works correctly for qMRI ${T}_{1}$ build-up curves, in analogy to our recent demonstrations of the method for ${T}_{2}$ relaxation or diffusion maps^20–22^. Hence, phasor plots enable unmixing multiple relaxation components in cardiac qMRI ${T}_{1}$ data without the need to use a fitting procedure. In addition, phasor processing is shown to enable visually unravelling *inter*-voxel correlations in cardiac qMRI ${T}_{1}$ data, such as partial-volume effects, that cannot be detected by per-voxel fitting methods. For the purpose of identifying partial-volume effects, both phasor plots and segmented phasor-coordinate images can be successfully used.

After this initial validation on cardiac data in the absence of MRI artifacts or disease, we have investigated the effect of simulated and measured motion on a qMRI ${T}_{1}$ dataset acquired from a healthy volunteer (Fig. 3). For a MOLLI experiment, motion causes per-voxel distortions in the signal recovery curves, because in such case the signal per voxel at each inversion time point originates from a different position in the body. As illustrated in Figs. 1 and 2 and in our previous works^20,21^, phasor offers a convenient way to directly visualize, within a single plot, signal characteristics that arise from all voxels in the image. The modelling results in Fig. 3b indicate that, with a simulated one-dimensional motion of about 9.3 mm, the scatter of datapoints perpendicular to the phasor reference curve increases by a factor of about 1.5 as compared to the same data without motion-induced shift. Similarly, the phasor plot in Fig. 3c shows an increase in datapoints scatter perpendicular to the phasor reference curve by a factor of about 2. We note that, in our previous work on the validation of full-harmonics phasor analysis^21^, a qualitatively similar increase in phasor datapoints scatter was observed for simulated bi-exponential data with added Gaussian noise, for which a thicker bi-exponential ‘line’ was observed than in the absence of noise, indicating an increased uncertainty in the estimate of the individual decay components. In the present case, due to the proximity of the bi-exponential cloud of datapoints to the phasor reference curve, the increase in data scattering appears perpendicular to the latter curve. In Fig. 3, the associated ${T}_{1}$ maps show some loss of resolution, markedly along the vertical spatial direction in Fig. 3e as expected from the simulated vertical shift of voxels (see Sect. “Measured and simulated motion-corruption effects”), but no further straightforward indication of motion. The $Axis1$ images (data not shown here) proved in agreement with the respective ${T}_{1}$ maps in Fig. 3, as also observed in Fig. 2 as well as in our previous applications of full-harmonics phasor^21^.

Our pilot study shows that motion artefacts cause deviations of the expected decay model in the form of increased data scattering in the phasor plots, perpendicularly to the bi-exponential ‘line’, as compared to respective artefact-free data. We note that such phasor assessment of motion artefacts relies on the acquisition of a reference motion-free image per volunteer, not always available. Also, such increased phasor cloud scattering effect must be further quantified against natural variations occurring across datasets. Upon these necessary validations, phasor may, in the future, be used for motion detection in qMRI acquisitions. The short phasor processing times may allow for seamless integration in a clinical workflow, potentially enabling the user to reacquire maps if motion artifacts are detected. Future research is warranted to investigate the clinical value of a phasor-based rapid motion quality assurance to aid robustness in clinical cardiac ${T}_{1}$ mapping.

We conclude that phasor representation, in the form of phasor plots and/or phasor-based images, is consistent with the information provided by single-exponential fitting. In addition, phasor plots can in the future potentially aid characterizing *inter-*voxel features and motion artifacts in qMRI ${T}_{1}$ data that cannot be easily, or at all in some cases, detected by visual inspection of conventional ${T}_{1}$ maps.

Based on the successful feasibility assessment of phasor processing for qMRI ${T}_{1}$ data from healthy volunteers, we have applied the same image analysis approach to inspect similar data from two patients with scarred myocardial tissue. Results in Fig. 4 further confirm that ${T}_{1}$ maps and phasor-coordinates images show similar results, and demonstrate that the scarred tissue can be identified in both representations. Remarkably, the phasor plots indicate that voxels involving either normal or scarred myocardial tissue cluster around neatly distinct areas with respect to phasor coordinates, i.e. with respect to the myocardial volume fraction. Specifically, in the examined case of a scar whose size is appreciable in conventional LGE and ${T}_{1}$ maps (see red arrows in Fig. 4), a third cluster of data points along the semicircle is unravelled in the phasor plot (red data points in Fig. 4d,i) for a scar tissue area, between the region interested by the healthy myocardium and that related to the blood pool. This third component arising from voxels within the ROI for scar tissue (see Figure S2 in SI) exhibits ${T}_{1}$ values longer than in remote myocardium approximately by a factor 1.2, in agreement with other literature results^27^. We note that $Axis1$ ranges are different, across the two investigated datasets in Fig. 4, for both phasor-coordinate images and respective phasor plots. The $Axis1$ range depends on the projection plane, chosen for each dataset to optimize the unmixing accuracy for multi-component qMRI decays, by maximizing the distance between the clusters of points along the reference curve, as well as the distance of the phasor cloud from the reference curve itself^21^. The $Axis1$ range may further vary across datasets because of inter-subject variability, of differences in the SNR and/or in the data sampling along the lifetime encoding, as in the case of Fig. 4. Although the projection plane is chosen, for each dataset, according to the same optimization strategy, and $Axis1$ phasor images prove consistent with the respective qMRI maps, quantitative inter-subject comparison in terms of the resulting $Axis1$ values is not possible.

Additionally we note that, for the patient data shown in Fig. 4, the phasor cloud of datapoints falls even closer to the reference curve than for data from healthy volunteers (see Fig. 2b). Thus, a substantial overlap is observed, within the measurement noise, between phasor datapoints arising from localised scar tissue and those from partial-volume effects throughout the entire image. In Fig. 4e,j the phasor-based image segmentation using $Axis1$ coordinate ranges, defined in the respective phasor plots of Fig. 4d,i, displays the location of phasor datapoints, attributed to blood, healthy or scar myocardial tissue, in the reconstructed image. In spite of partial overlap of blue and red phasor datapoints in the ROI definition in Fig. 4d,i, for both patient data healthy myocardium and scar tissue can be separated, most clearly in Fig. 4j, as also suggested by the respective ${T}_{1}$ and phasor coordinate image (Fig. 4g,h). In agreement with the respective phasor plots, partial volume effects in these segmentation images arise from phasor datapoints too close to the reference curve to be separated from scar tissue voxels. We note that a different choice of ROIs in the phasor plots, involving also the use of $Axis2$ coordinate, could potentially aid better separating healthy and scar myocardial tissue image voxels, but would not remove such overlap with partial-volumed voxels.

Yet, in this pilot study we find that phasor holds potential for future use for segmentation of ${T}_{1}$ qMRI datasets and detection of scarred myocardial tissue in patient data, with the aid of algorithm-assisted full-harmonics projection and phasor-based segmentation. The data examined in this feasibility study were acquired without the use of contrast agents and processed without resorting to any fitting procedure or assumption on the number or values of ${T}_{1}$ lifetime components. We add that phasor approach can be of special interest for cases where the mono-exponential model is by definition not applicable. Examples of these systems are quadrupolar nuclei, where both the ${T}_{1}$ and ${T}_{2}$ relaxation models are multiexponential by nature, or systems with susceptibility enclosures such as the lungs or the vicinity of blood vessels where the transverse magnetization decay has been shown to deviate from a mono-exponential decay^28,29^. Finally, due to the complex nature of biological tissue, the accuracy of the phenomenological Bloch equations in those systems has also been challenged under certain circumstances^30,31^. Thus, a model-free evaluation may generally yield additional insights especially if highly accurate measurement techniques are being used. Finally, the computational ease and independence from fitting procedures of phasor can be utilized for the ever-increasing efforts towards optimizing the use of machine learning algorithms for the analysis of big qMRI data.

## Conclusions

In this work, we have evaluated the feasibility of phasor processing method for the analysis of cardiac ${T}_{1}$ mapping data. Our data indicate the potential use of phasor analysis, in the form of phasor-coordinate maps, phasor plots and/or phasor-based segmentation images, for the eased depiction of partial volume effects and motion, while retaining good visualization of abnormal relaxation times in patients. Hence, phasor may offer potential as an alternative, model-free, qMRI data analysis method for fast and robust tissue characterization based on conventional myocardial ${T}_{1}$ mapping data. Future research is warranted to further validate the clinical value of a phasor-based quality assurance to aid robustness in clinical cardiac ${T}_{1}$ mapping of motion artefacts and scar tissue.

## Supplementary Information

Supplementary Information.

## Data Availability

The datasets generated during the current study are available from the corresponding author on reasonable request. The description of the code used for full-harmonics phasor analysis is described in detail in our previous publication: https://pubs.acs.org/doi/pdf/10.1021/acs.jpclett.0c02319.

## Abbreviations

qMRI: Quantitative MRI
MOLLI: Modified look-locker inversion recovery
FT: Fourier transform
ROI: Region-of-interest
LGE: Late gadolinium enhanced
