# Supplementary material for: Model-free phasor image analysis of quantitative myocardial T1 mapping
Source: Sci Rep. 2022 Nov 18;12:19840. doi: 10.1038/s41598-022-23872-9 (PMC9674690; doi:10.1038/s41598-022-23872-9)
Supplement: Supplementary file 1 — Supplementary Information. [file 41598_2022_23872_MOESM1_ESM.docx]

Supplementary Information for:

Model-free phasor image analysis of quantitative myocardial T_1_ mapping

Wouter M. J. Franssen^1^, Thomas Treibel^2^, Andreas Seraphim^2^, Sebastian Weingärtner^3^, Camilla Terenzi^1,*^

^1^Laboratory of Biophysics, Wageningen University & Research, Wageningen, The Netherlands.

^2^Institute of Cardiovascular Science, University College London, London, UK. Department of Cardiology, St Bartholomew's Hospital, Barts Health NHS Trust, London, UK.

^3^Department of Imaging Physics, Delft University of Technology, Delft, The Netherlands.

*Corresponding author: [camilla.terenzi@wur.nl](mailto:camilla.terenzi@wur.nl)

# CHOICE OF PROJECTION PLANE IN full-harmonics PHASOR ANALYSIS

For an exponential decay sampled with an even number $N$ of real data points, the Fourier series contains a maximum of $N/2+1$ harmonics. In phasor analysis, the intensity of the zeroth-order harmonic is used as a normalization value for all other harmonics. The remaining $N/2$ harmonics, with both a real and imaginary part, yield an $N$-dimensional phasor space and can be used to construct the full-harmonics phasor plots.^1^

The definition of a projection plane for full-harmonics phasor analysis requires the selection of two orthogonal $x$ and $y$ axes, hereby respectively indicated as $\overline{\mathrm{Axis}1}$ and $\overline{\mathrm{Axis}2}$. As shown already in our previous paper,^1^ this can be achieved by choosing three points in the $N$-dimensional phasor space of the measured dataset, with $N$ corresponding to the number of real data points used for sampling the exponential decay. In order to facilitate the selection of these phasor coordinate points, we consider only phasor coordinates along the semicircle, from which lifetime values can directly be calculated. In this way, one needs to select three distinct lifetimes to define the full-harmonics projection plane. Two lifetimes are chosen so as to correspond to either the shortest or the longest lifetime value expected in the dataset. A third lifetime value is chosen in between these two values.

Based on the phasor coordinates of these three lifetimes in the $N$-dimensional phasor space, given by the vectors $\overline{y1}$, $\overline{y2}$ and $\overline{y3}$, we can calculate the full-harmonics phasor projection axes by following in order the steps indicated below:

1. Definition of $\overline{v1}$ as the difference between the phasor coordinates for the shortest and the longest lifetime, respectively $\overline{y1}$ and $\overline{y3}$, yielding: $\overline{v1}=\overline{y1}-\overline{y3}$.
2. Normalization of $\overline{v1}$ to obtain $\overline{\mathrm{Axis}1}$, yielding $\overline{\mathrm{Axis}1}= \frac{\overline{v1}}{\left| \overline{v1} \right|}=\left\| \overline{v1} \right\|$.
3. Definition of $\overline{v2}$ as the difference between the phasor coordinates for the shortest and the intermediate lifetime, respectively $\overline{y1}$ and $\overline{y2}$, according to: $\overline{v2}=\overline{y1}-\overline{y2}$.
4. Gram-Schmidt orthogonalization of $\overline{v2}$ with respect to $\overline{\mathrm{Axis}1}$ by the following steps:
   1. Calculation of the projection of $\overline{v2}$ on $\overline{\mathrm{Axis}1}$: $\overline{\mathrm{err}}=\overline{v2}\bullet\overline{\mathrm{Axis}1}$, where the symbol “$\bullet$” indicates the inner product.
   2. Subtraction of the above projection from $\overline{v2}$ to obtain $\overline{u2}$ as $\overline{u2}=\overline{v2}-\overline{\mathrm{err}}$.
   3. Normalization of $\overline{u2}$ to obtain $\overline{Axis2}$, yielding $\overline{Axis2}=\left\| \overline{u2} \right\|$.

The selected $\overline{\mathrm{Axis}1}$ and $\overline{\mathrm{Axis}2}$ values can now be used for constructing the full-harmonics phasor plot. Given a generic phasor position $\overline{P}$, its coordinates along the chosen axes are simply calculated using the inner product, respectively as $\overline{P}\bullet\overline{Axis1}$ or $\overline{P}\bullet\overline{Axis2}$.

For pure visualization purposes, in most cases $\overline{Axis1}$ and $\overline{Axis2}$ must be rotated to make the resulting plot more similar to the usual, first harmonic, phasor plot.^1^ This is obtained by imposing that (i) all phasor coordinates must be positive along the two phasor plot axes, and that (ii) ideally, in the case of exponential decays devoid of noise, all data points must fall within the phasor plot area defined by the semicircle and the rotated $\overline{Axis1}$. To this scope, both edges of the reference semicircle must lie along the positive $x-$axis. In some cases, a flip of the $y$-axis is also needed to get the semicircle in the $y > 0$ region.

We refer to Figure S2 of the Supplementary Information in our previous paper^1^ for a visualization of the projection plane, possible in the case of $N=3$.

# SUPPLEMENTARY Figures


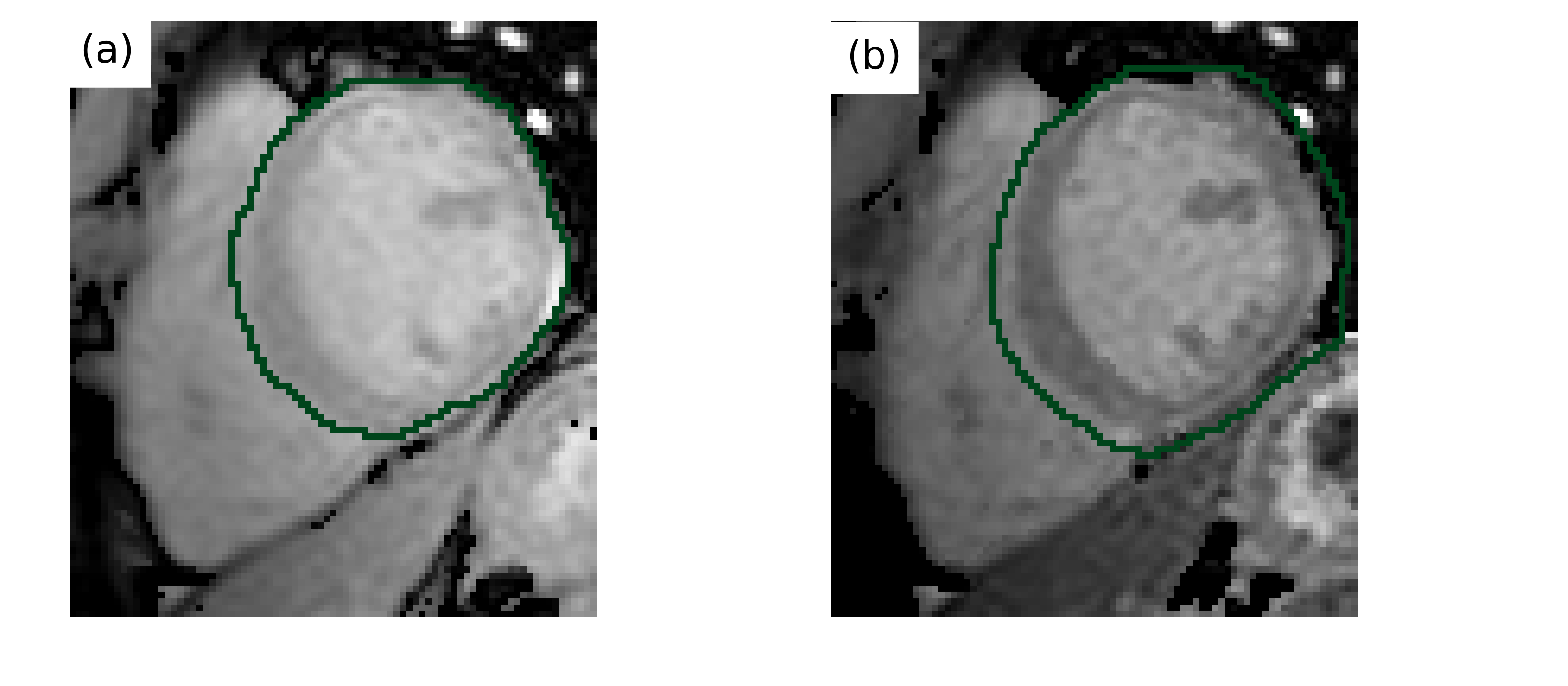
Figure S1: ^1^H MRI intensity maps of the two data sets with motion artefacts shown in Figure 3, with the selected ROI for phasor analysis outlined in green. (a): for the breath-hold and simulated motion data (Figure 3a,d and 3b,e). (b): for the free-breathing data (Figure 2c,f).


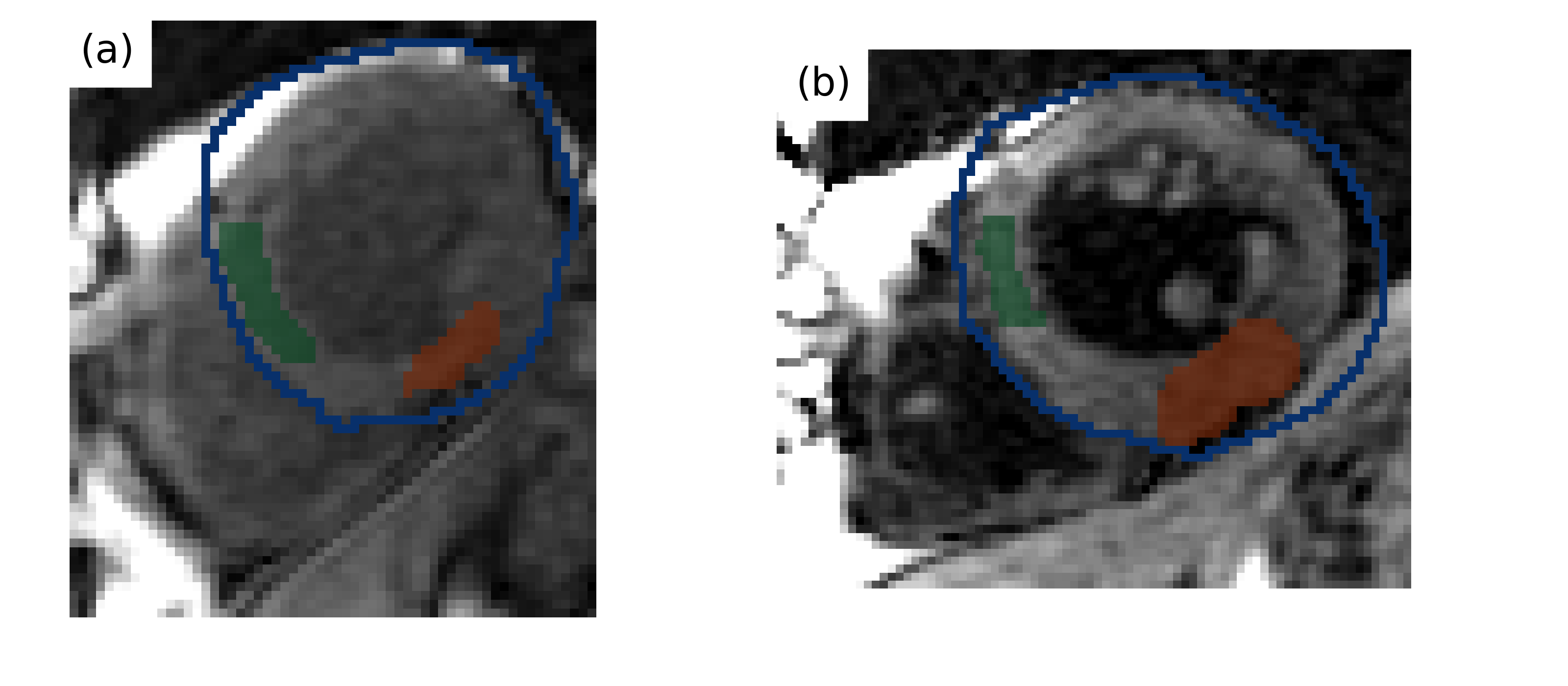
Figure S2: ^1^H MRI intensity maps for the two patient datasets shown in Figure 4, showing the scar (brown) and normal (green) region of interest for (a) the top row of Figure 4, (b) the bottom row of Figure 4. The selected ROI for phasor analysis is outlined in blue.

# References

1 Franssen, W. M. J., Vergeldt, F. J., Bader, A. N., van Amerongen, H. & Terenzi, C. Full-Harmonics Phasor Analysis: Unravelling Multiexponential Trends in Magnetic Resonance Imaging Data. *J. Phys. Chem. Lett.* **11**, 9152-9158, (2020).
